# The Italian registry of pulmonary non-tuberculous mycobacteria - IRENE: the study protocol

**DOI:** 10.1186/s40248-018-0141-8

**Published:** 2018-08-09

**Authors:** Stefano Aliberti, Luigi Ruffo Codecasa, Andrea Gori, Giovanni Sotgiu, Maura Spotti, Antonio Di Biagio, Andrea Calcagno, Stefano Nardini, Baroukh Maurice Assael, Enrico Tortoli, Giorgio Besozzi, Maurizio Ferrarese, Alberto Matteelli, Enrico Girardi, Saverio De Lorenzo, Manuela Seia, Andrea Gramegna, Bruno Del Prato, Leonardo Terranova, Martina Oriano, Nicola Sverzellati, Mehdi Mirsaeidi, James D. Chalmers, Charles S. Haworth, Michael R. Loebinger, Timothy Aksamit, Kevin Winthrop, Felix C. Ringshausen, Giuliana Previdi, Francesco Blasi, on behalf of the IRENE Network, Giuliana Fusetti, Manuela Martorana, Gennaro Colella, Giuseppina Verga, Gerardina Carbone, Isabella Damilano, Fabrizio Nava, Lisa Pancini, Ana Pasat, Nicolò Vanoni

**Affiliations:** 1 Department of Pathophysiology and Transplantation, University of Milan, Via Francesco Sforza 35, 20122 Milan, Italy; 2 Internal Medicine Department, Respiratory Unit and Cystic Fibrosis Adult Center, Fondazione IRCCS Ca’ Granda Ospedale Maggiore Policlinico, Via Francesco Sforza 35, 20122 Milan, Italy; 3 Regional TB Reference Centre, Istituto Villa Marelli, ASST Grande Ospedale Metropolitano Niguarda, Milan, Italy; 4 Clinic of Infectious Diseases, ‘San Gerardo” Hospital-ASST Monza, University Milano-Bicocca, Milan, Italy; 5 Clinical Epidemiology and Medical Statistics Unit, Department of Clinical and Experimental Medicine, University of Sassari, Sassari, Italy; 6 Clinica Malattie Infettive, Policlinico Ospedale S. Martino, Genoa, Italy; 7 Unit of Infectious Diseases, Department of Medical Sciences, University of Torino, Torino, Italy; 8 Ospedale Civile, Pulmonary and TB Unit, Vittorio Veneto, Italy; 9 Emerging Bacterial Pathogens Unit, IRCCS San Raffaele Scientific Institute, Milan, Italy; 10 StopTB Italia, Milan, Italy; 11 WHO Collaborating Centre for TB/HIV co-infection and TB Elimination, Department of Infectious and Tropical Diseases, University of Brescia, Brescia, Italy; 12 Clinical Epidemiology Unit, National Institute for Infectious Disease “L. Spallanzani, Rome, Italy; 13 E. Morelli Hospital ASST, Reference Center for MDR-TB and HIV-TB, Sondalo, Italy; 14 Medical Genetics Laboratory, Fondazione IRCCS Ca’ Granda Ospedale Maggiore Policlinico, Milan, Italy; 15 Unit of Interventional Pulmonology, High Speciality “A. Cardarelli” Hospital, Naples, Italy; 16 Pediatric Highly Intensive Care Unit, Department of Pathophysiology and Transplantation, Fondazione IRCCS Ca’ Granda Ospedale Maggiore Policlinico, Università degli Studi di Milano, Milan, Italy; 17 Scienze Radiologiche, Dipartimento di Medicina e Chirurgia, Università di Parma, Parma, Italy; 18 Miami Veterans Administration Medical Center, Division of Pulmonary, Allergy, Critical Care, and Sleep Medicine, University of Miami School of Medicine, Miami, FL USA; 19 Scottish Centre for Respiratory Research, University of Dundee, Ninewells Hospital and Medical School, Dundee, UK; 20 Cambridge Centre for Lung Infection, Papworth Hospital, Cambridge, UK; 21 Host Defence Clinic, Royal Brompton Hospital, London, UK; 22 Mayo Clinic College of Medicine, Rochester, MN USA; 23 Oregon Health and Science University, Portland, OR USA; 24 Dept of Respiratory Medicine, Member of the German Centre for Lung Research, Hannover Medical School, Hannover, Germany; 25 Aziende Socio Sanitarie Territoriale Melegnano e della Martesana, Vizzolo Predabissi, Milan, Italy

**Keywords:** NTM, NTM-PD, COPD, *M. Avium*, *M. Intracellulare*, Atypical mycobacteria, Bronchiectasis, Cystic fibrosis, Lung transplant, HIV

## Abstract

**Background:**

A substantial increase in pulmonary and extra-pulmonary diseases due to non-tuberculous mycobacteria (NTM) has been documented worldwide, especially among subjects suffering from chronic respiratory diseases and immunocompromised patients. Many questions remain regarding the epidemiology of pulmonary disease due to NTM (NTM-PD) mainly because reporting of NTM-PD to health authorities is not mandated in several countries, including Italy. This manuscript describes the protocol of the first Italian registry of adult patients with respiratory infections caused by NTM (IRENE).

**Methods:**

IRENE is an observational, multicenter, prospective, cohort study enrolling consecutive adult patients with either a NTM respiratory isolate or those with NTM-PD. A total of 41 centers, including mainly pulmonary and infectious disease departments, joined the registry so far. Adult patients with all of the following are included in the registry: 1) at least one positive culture for any NTM species from any respiratory sample; 2) at least one positive culture for NTM isolated in the year prior the enrolment and/or prescribed NTM treatment in the year prior the enrolment; 3) given consent to inclusion in the study. No exclusion criteria are applied to the study. Patients are managed according to standard operating procedures implemented in each IRENE clinical center. An online case report form has been developed to collect patients’ demographics, comorbidities, microbiological, laboratory, functional, radiological, clinical, treatment and outcome data at baseline and on an annual basis. An IRENE biobank has also been developed within the network and linked to the clinical data of the registry.

**Conclusions:**

IRENE has been developed to inform the clinical and scientific community on the current management of adult patients with NTM respiratory infections in Italy and acts as a national network to increase the disease’s awareness.

**Trial registration:**

Clinicaltrial.gov: NCT03339063.

## Background

Pulmonary disease due to non-tuberculous mycobacteria (NTM) has always been a tangible clinical entity [1]. An increase in NTM pulmonary and extra-pulmonary morbidity and mortality has been documented in Italy and worldwide, especially among patients suffering from chronic respiratory diseases, including bronchiectasis, chronic obstructive pulmonary disease (COPD), or cystic fibrosis (CF), as well as among HIV-positive and other immunocompromised patients [2–11]. Several determinants of the increasing epidemiological trend have been identified: the aging of the population with high prevalence of chronic and debilitating diseases; an intensified use of immunosuppressive therapies; a broader use of chest CT; a high diagnostic yield of microbiological conventional and molecular techniques; an increasing environmental exposure to NTM; an increase use of antibiotics which can favor the occurrence of niches for NTM; declining rates of *M. tuberculosis* infection; and a potential impact of person-to-person transmission as recently suggested among CF patients [12, 13].

Many questions remain regarding the epidemiology of pulmonary disease due to NTM (NTM-PD), which is characterized by symptomatic, progressive inflammatory lung damage and defined in 2007 in the American Thoracic Society (ATS) / Infectious Diseases Society of America (IDSA) guidelines [14, 15]. These epidemiological and clinical uncertainties on NTM-PD cause significant confusion for clinicians in daily clinical practice when asked to diagnose NTM-PD. The clinical relevance of specific NTM respiratory isolates significantly varies from patient to patient, and the interplay between exposure- and host-related factors is poorly understood [15]. NTM-PD shows a wide spectrum of clinical manifestations and frequently is diagnosed in the context of concomitant respiratory diseases (e.g., bronchiectasis, COPD, or CF) [3]. Geographical diversity is another important factor in the epidemiology of NTM. A large inter- and intra-country heterogeneity in distribution of NTM species has been recently shown [16, 17]. Finally, an Italian experience described NTM-PD risk factors whose qualitative and quantitative ascertainment could help clinicians to discriminate between colonization and disease [18].

Reporting of NTM-PD to health authorities is not mandated in several countries and the current estimates have been obtained from sentinel surveillance or laboratory-based studies, retrospective cohort studies, or audits of administrative databases. Only few European countries (i.e., UK, Greece, Germany, and the Netherlands) have provided epidemiological data, showing an incidence rate of NTM isolation ranging from 2.9 to 7.0 per 100,000 population and a NTM-PD prevalence of 0.7–1.7 per 100,000 population, with a marked increase as aging occurs [19–21]. Until now no data have been published on the epidemiology of respiratory NTM infections in Italy.

On this basis, robust national longitudinal data are needed. This manuscript describes the protocol of the first Italian registry of adult patients with respiratory infections caused by NTM.

## Methodology of the Irene registry

### Study design

**Fig. 1:**
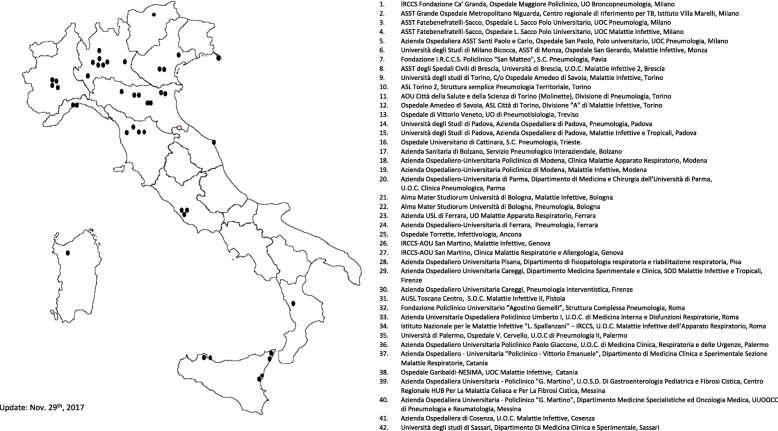
The IRENE study site

The Italian registry of pulmonary NTM (IRENE) is an observational, multicenter, prospective, cohort study enrolling consecutive adult patients with either a NTM respiratory isolate or those with NTM-PD. The coordinating center is located at the Pulmonary Department of the Fondazione IRCCS Ca′ Granda, Ospedale Maggiore Policlinico (hereby referred to as Policlinico Hospital), Milan, Italy, where the central approval from the Ethical Committee (EC) for this study was obtained on March 6th, 2017, and the first patient was enrolled on April 21st, 2017. A total of 42 centers, including mainly pulmonary and infectious disease (ID) departments, joined the registry so far, see Fig. 1. All the centers are also required to obtain local EC approval before entering the registry. All patients must provide written informed consent to participate in the registry. The study is sponsored by the Policlinico Hospital in Milan. The study website is located at www.registroirene.it and the study has been registered at clinicaltrial.gov (NCT03339063).

### Study subjects

Adult (≥18 years) patients with all of the following are included in the registry: 1) at least one positive culture for any NTM species from any respiratory sample; 2) at least one positive culture for NTM isolated in the year prior the enrolment and/or prescribed NTM treatment in the year prior the enrolment; 3) given consent to inclusion in the study. No exclusion criteria are applied to the study in order to increase the generalizability of the results. The inclusion criteria of the registry clearly identify a population of patients characterized by a recent/ongoing history of either a NTM infection or NTM-PD (Table 1). Patients who received NTM treatment in the year prior to enrolment but did not have a positive culture for NTM isolated in this period of time could be included in the study if still they had at least one positive culture for any NTM species from any respiratory sample in their history. The IRENE Executive Committee decided not to limit the enrolment to patients with NTM-PD, but to follow up also patients with a recent NTM infection not fulfilling the 2007 ATS/IDSA criteria for NTM-PD [15].

**Table 1:** IRENE inclusion and exclusion criteria

Inclusion criteria (all of them)	
• Adults (≥18 years)	
• Any ethnicity	
• Any gender	
• At least one positive culture for any non-tuberculous mycobacteria species from any respiratory sample	
• At least one positive culture for non-tuberculous mycobacteria isolated in the year prior the enrolment and/or prescribed treatment for non-tuberculous mycobacteria in the year prior the enrolment	
• Given consent to inclusion in the study	
Exclusion criteria	
• None	

Patients included in the registry are mainly recruited among pulmonary and ID out- and in-patient services. Adult CF, lung transplant, and tuberculosis clinics represent other recruitment centers. A heterogeneous population of patients with NTM infection/NTM-PD sharing different clinical phenotypes is expected to be enrolled in the registry. IRENE has a special focus on four patients’ categories: 1) immunocompetent/bronchiectatic, 2) HIV-positive, 3) CF and 4) lung transplanted patients. 500 patients are expected to be enrolled in the registry by the end of 2020. The registry has been developed to accept an unlimited number of patients and no deadlines have been decided.

### Data collection, definitions and quality control

Patients are managed according to standard operating procedures (SOPs) implemented in each IRENE clinical center without any interference from the study team. A baseline case report form (CRF) is collected at patient’s enrolment including demographics, comorbidities, microbiological, laboratory, functional, radiological, clinical, and treatment data. Then, study investigators will enter follow-up data on an annual basis. Furthermore, a “start treatment” and a “stop treatment” CRFs are also collected. The database incorporates automated logic checks put in place to avoid the collection of out-of-range values. Once the case is entered into the registry, two members of the study team (SA and MS) manually verified its consistency and data queries will be solved with the local study investigator. In case of unresolved queries or incomplete cases, they will be rejected to have high quality data. To assure the high quality of the data, random audit will also be conducted at study sites.

### IRENE biobank

An IRENE biobank has also been developed within the network and linked to the clinical data of the registry. IRENE sites can collect samples, including blood, serum, plasma, respiratory specimens (e.g., sputum, induced sputum, tracheal aspirate, or bronchoalveolar lavage), urine, and NTM isolates at the first visit and during follow-up on a voluntary basis. The same SOPs for biological collection, processing, and storage (first locally and then centralized at the Policlinico Hospital in Milan for analysis) will be adopted by all IRENE sites.

### The registry governance and the IRENE network

The registry is held securely at the Health Informatics Centre (HIC) of the Policlinico Hospital in Milan, Italy, and de-identified data will be accessible to the principal investigator and to all IRENE investigators on request to the Executive Committee. Analysis of the entire IRENE database will be allowed after the submission of a research question, along with a specific study protocol, to the IRENE Scientific Committee. IRENE investigators will have unrestricted access to their own data. The database will be run in accordance with the principles of Good Clinical Practice. Study results will be disseminated in the form of annual reports, conference abstracts, and peer reviewed publications. The IRENE network will follow the International Committee of Medical Journal Editors recommendations regarding authorship.

IRENE is the official Italian network within the EMBARC European NTM registry [22]. The EMBARC (European Multicentre Bronchiectasis Audit and Research Collaboration) registry is a prospective, pan-European observational study of adult patients with bronchiectasis, including those with NTM. Patients enrolled in the EMBARC registry undergo a comprehensive baseline assessment and are followed-up annually for up to 5 years with the goal of providing high-quality longitudinal data on outcomes, treatment patterns and quality of life [22]. All Italian patients included in the EMBARC European NTM registry will be enrolled through IRENE and IRENE data will be incorporated into the EMBARC European NTM registry.

IRENE also promotes multi-disciplinary education and patient-professional collaboration in the field of NTM through its relationship with national scientific societies. There is a lack of a platform for communication between patients with NTM infection/NTM-PD and physicians in Italy and some patients within the IRENE network already expressed their will to develop a patient advisory group.

### The NTM Consilium

Inspired by recent successful initiatives supporting physicians in their decisions to manage difficult-to-treat patients with respiratory infection, such as the ERS/WHO TB Consilium, a free-cost, Italian, interned-based consultation system will be developed within the IRENE website, the IRENE NTM Consilium [23]. IRENE investigators will have access to this platform to seek advice from national experts on the clinical management of complicated NTM cases and will receive a suggestion in less than 3 working days. A call for experts will precede the launch of this initiative.

## Discussion

Several registries and merging platforms for international cohorts have been developed on different respiratory diseases, including community-acquired pneumonia (the CAPO database), bronchiectasis (EMBARC and FRIENDS), or primary ciliary dyskinesia over the past decades [24, 25]. Registries are invaluable tools which can help investigators to better understand the natural history, the epidemiology, and the management of a specific disease [26]. Results from the registries may inform both the clinical community on real-life data and the scientific experts on tracks for future interventional studies.

The largest registry including NTM patients running so far is the United States Bronchiectasis Research Registry (BRR) which is a database sponsored by the COPD Foundation and which has enrolled 1826 non-CF bronchiectasis patients at 13 sites in the USA until now [27]. The first report from the BRR has been recently published showing 63 % of the population with a history of NTM disease or NTM isolation [27]. Important differences between bronchiectasis patients with and without NTM were identified by the authors. Although extremely informative, this registry has been mainly developed as a bronchiectasis registry with some specific data on NTM-PD. IRENE may integrate BRR data because it includes > 40 sites where NTM patients are cared by both pulmonary and ID physicians to fully explore the disease heterogeneity.

IRENE has been developed: 1) to strengthen the network of pulmonologists, infectious diseases physicians, and other healthcare providers caring for adult patients with NTM; 2) to prospectively collect demographics, clinical, microbiological, radiological, functional, and therapy variables, as well as long-term outcomes; 3) to longitudinally collect clinical, biological and mycobacteriological samples for future translational research on NTM; 4) to assess the heterogeneity in the clinical manifestation of the disease and its current management; 5) to release national SOPs and guidelines based on precise epidemiological data; 4) to increase NTM disease awareness at national and international levels. The IRENE network is composed by pulmonologists, ID physicians, clinical microbiologists, tuberculosis, CF and lung transplant specialists, radiologists, public health experts, translational researchers, an international advisory board of NTM experts, as well as patients suffering from NTM infections. IRENE has been designed to be open for collaboration with other national and international registries in the field of NTM. The IRENE data fields and protocol are aligned with the European NTM registry to ensure data can be shared between IRENE and the European NTM Registry for collaborative analysis [22].

## Conclusions

NTM-PD is considered a neglected and rare disease and there are limited observational and experimental data on epidemiology of this condition and its management. A substantial improvement in the understanding of NTM infection and NTM-PD is needed to develop new therapies and improve patients’ outcomes. IRENE has been developed to inform the clinical and scientific community on the current management of adult patients with NTM respiratory infections in Italy and acts as a national network to increase the disease’s awareness.

## Abbreviations

ATS: American Thoracic Society
BRR: Bronchiectasis Research Registry
CAPO: Community-Acquired Pneumonia Organization
CF: Cystic fibrosis
COPD: Chronic Obstructive Pulmonary Disease
CRF: Case Report Form
CT: Computed Tomography
EC: Ethical Committee
EMBARC: European Multicentre Bronchiectasis Audit and Research Collaboration
FRIENDS: Facilitating Research Into Existing National DataSets
HIC: Health Informatics Centre
HIV: Human Immunodeficiency Virus
ID: Infectious Disease
IDSA: Infectious Diseases Society of America
IRCCS: Istituto di Ricovero e Cura a Carattere Scientifico
IRENE: The Italian REgistry of pulmonary Non-tuberculous mycobactEria
IRS: Italian Respiratory Society
NTM: Non-Tuberculous Mycobacteria
NTM-PD: pulmonary disease due to NTM
PCD: Primary Ciliary Dyskinesia
SOPs: Standard Operating Procedures
TB: Tuberculosis
UK: United Kingdom
